# Elevated CD147 expression is associated with shorter overall survival in non-small cell lung cancer

**DOI:** 10.18632/oncotarget.16948

**Published:** 2017-04-07

**Authors:** Xiaojun Zhang, Tian Tian, Xiaofeng Zhang, Changting Liu, Xiangqun Fang

**Affiliations:** ^1^ Nanlou Respiratory Diseases Department, Chinese PLA General Hospital, Beijing 100853, P. R. China

**Keywords:** CD147, lung cancer, meta-analysis, prognosis, survival

## Abstract

A number of studies have reported on the prognostic role of CD147 expression in non-small cell lung cancer (NSCLC); however, the results remain controversial. This study aims to investigate the impact of CD147 on the prognosis of NSCLC by means of a meta-analysis. A literature search was performed for relevant studies published before October 29, 2016. The hazard ratios (HRs), odds ratios (ORs), and 95% confidence intervals (CIs) were calculated as effective measures. Sensitivity analysis and publication bias examination were also conducted. Ten eligible studies with a total of 1605 patients were included in this meta-analysis. CD147 overexpression was correlated with poor overall survival (OS) (HR=1.59, 95% CI=1.32–1.91, p<0.001). Elevated CD147 expression was associated with the presence of lymph node metastasis (OR=2.31, 95% CI=1.74–3.07, p<0.001) and advanced TNM stage (OR=3.03, 95% CI=1.24–7.39, p=0.015). However, no significant association between CD147 and sex, age, differentiation, or histology was found. No evidence of significant publication bias was identified. This meta-analysis revealed that overexpression of CD147 was associated with shorter OS, the presence of lymph node metastasis and advanced TNM stage in NSCLC. Therefore, CD147 could serve as a potential prognostic marker for NSCLC.

## INTRODUCTION

Lung cancer remains the leading cause of cancer mortality globally [1]. In the US, it is estimated that 222,500 new lung cancer cases will be diagnosed and 155,870 deaths will occur due to this disease in 2017 [2]. Lung cancer is histologically categorized into two types: non-small cell lung cancer (NSCLC) and small cell lung cancer (SCLC). NSCLC accounts for 85% of all lung cancer cases [3]. It is known that several prognostic factors including advanced stage, poor differentiation, and distant metastasis could be indicators for poor clinical outcomes [4, 5], however the prognosis for NSCLC is still poor [6]. The identification of novel and more effective biomarkers is urgently required for prognostication in NSCLC.

CD147 (also termed extracellular matrix metalloproteinase inducer, EMMPRIN, basigin, or HAb18G) is a member of the immunoglobulin family [7]. CD147 is widely expressed on the cell surface of different cancer types [8]. CD147 has been demonstrated to facilitate the synthesis and secretion of matrix metalloproteinases (MMPs)[9]. CD147 can also degrade the basement membrane and consequently promote tumor invasion. It is reported that CD147 is highly expressed in various malignant tumors and is associated with survival outcomes [10–16]. Previous studies also investigated the relationship between CD147 and NSCLC prognosis, however, those results were controversial [17–26]. Because of the limitations of small sample size and heterogeneous detection methods in these studies, a quantitative evaluation of these conflicting data is necessary. We therefore performed a meta-analysis to explore the association between CD147 and the prognosis and clinicopathological features in NSCLC.

## RESULTS

### Eligible studies and characteristics

**Figure 1:**
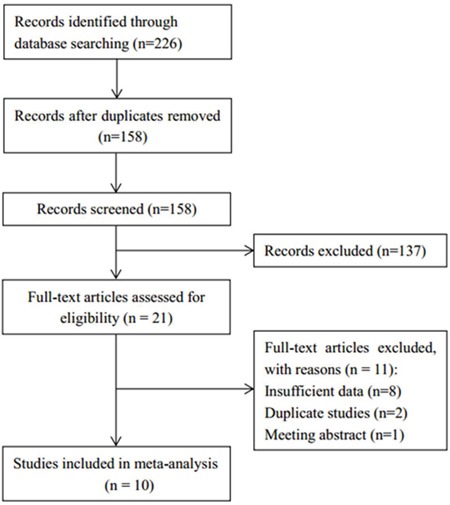
Flow chart of study selection

The selection process for eligible studies is presented in Figure 1. An electronic database search obtained a total of 226 records. Following the removal of duplications, 158 records were obtained. Through examination of the title and abstract, 137 records were excluded because they did not meet the inclusion criteria or were irrelevant studies. Following a review of the remaining 21 full-text articles, 11 studies were further excluded because of insufficient data (n=8), duplicate studies (n=2), and meeting abstract (n=1). Therefore, 10 eligible studies [17–26] were included in the final meta-analysis. The baseline characteristics of the included studies are shown in Table 1. The total sample size was 1605, with a range from 55-327. Seven studies [18–20, 22, 24–26] were published in English and 3 studies [17, 21, 23] were in Chinese. Eight studies [18, 19, 21–26] presented data on the relationship between CD147 and overall survival (OS) and 8 studies [17, 19–21, 23–26] reported the association between CD147 and clinical factors. All studies had Newcastle-Ottawa scale scores ≥6.

**Table 1:** Baseline characteristics of included studies

Study	Year	Country	Patients(n)	Gender(M/F)	Age (years)mean (range)	Detectionmethod	Stage	Cutoff	Positive(%)	NOS
Dong	2005	China	112	83/29	59.6(30-79)	IHC	I-III	>50%	62.5	7
Fei	2014	China	241	183/58	59	IHC	I-III	>50%	82.2	6
Hakuma	2007	Japan	208	139/69	NA	IHC	I-IV	>25%	92	7
Kefeli	2010	Turkey	64	59/5	60.1	IHC	I-IV	>25%	96	7
Liu	2010	China	327	269/68	59(29-82)	IHC	I-IV	>50%	67	8
Sienel	2008	Germany	150	115/35	NA	IHC	I-III	2*	41	7
Wang	2011	China	55	34/21	57.3 (25-81)	IHC	I-III	>25%	61.8	7
Xu	2013	China	150	110/40	60(35-82)	IHC	I-IV	>30%	82.7	8
Zeng	2011	China	118	102/16	60	IHC	III-IV	>25%	85.6	8
Zhong	2013	China	180	123/57	60(37-75)	IHC	I	>25%	62.2	7

NA: not available; IHC: immunohistochemical staining; NOS: Newcastle-Ottawa scale.

*Score 2 means intensity equal to positive control in Ref [22].

### Association between CD147 and OS

**Figure 2:**
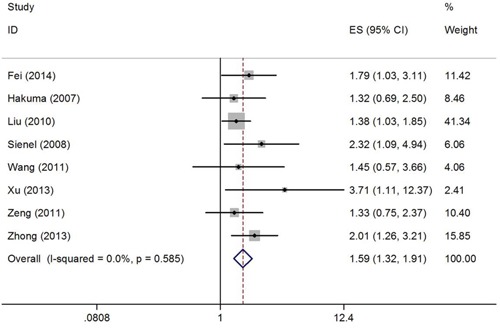
Forest plot of HR was assessed for association between CD147 and OS in NSCLC

Hazard ratios (HRs) and 95% confidence intervals (CIs) regarding CD147 and OS were extracted from a total of 8 studies [18, 19, 21–26] involving 1429 patients. Because of no significant between-study heterogeneity (*I*^2^=0%, P_h_=0.585), the fix effect model was used to calculate the pooled HR. The results suggested that CD147 overexpression was correlated with poor OS (HR=1.59, 95% CI=1.32–1.91, p<0.001). The results are presented as a forest plot in Figure 2.

### Relationship between CD147 and clinicopathological characteristics

**Figure 3:**
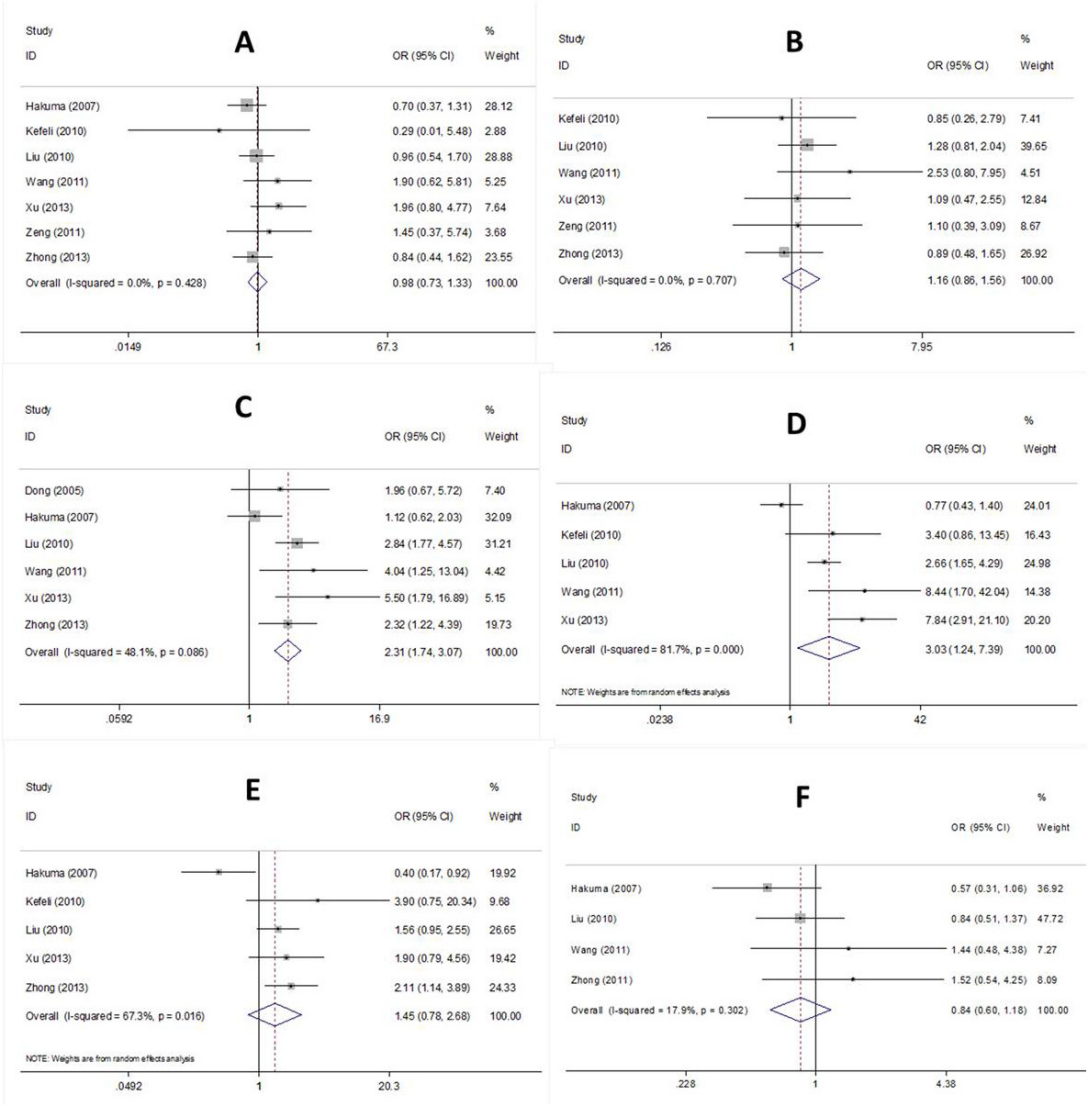
Forest plot of CD147 associated with **(A)** sex, **(B)** age, **(C)** LN metastasis, **(D)** TNM stage, **(E)** differentiation, and **(F)** histology in NSCLC patients

The associations between CD147 and six clinicopathological factors were investigated. The relevant factors were sex, age, lymph node metastasis, TNM stage, differentiation, and histology. The pooled ORs and 95% CIs are summarized in Figure 3. The pooled data demonstrated that elevated CD147 expression was associated with the presence of lymph node metastasis (OR=2.31, 95% CI=1.74–3.07, p<0.001) and advanced TNM stage (OR=3.03, 95% CI=1.24–7.39, p=0.015). However, no significant connection between CD147 and sex, age, differentiation, or histology was found.

### Sensitivity analysis

**Figure 4:**
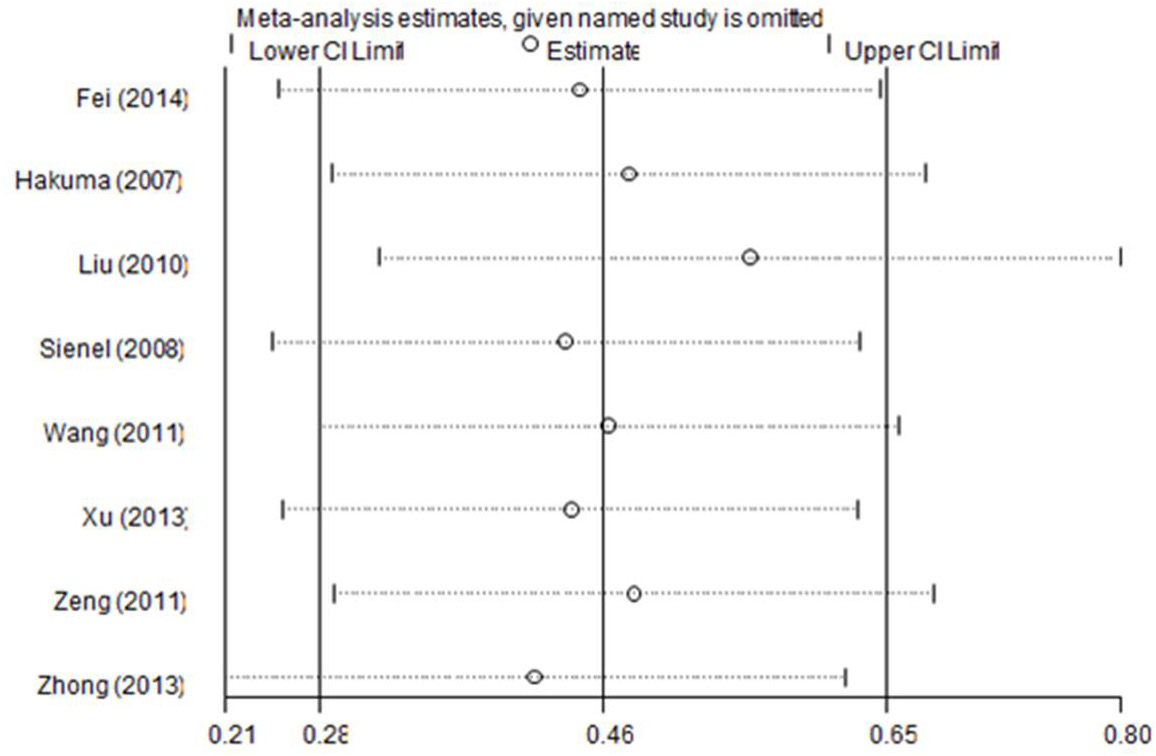
Sensitivity analyses of the summary HR for OS

To examine the robustness of the results, sensitivity analysis by sequential omission of each included study was conducted. As shown in Figure 4, the pooled HR and 95% CI were not substantially altered, which indicated the credibility of our results.

### Publication bias

Begg's test was applied to examine potential publication bias, and no obvious publication bias was found in OS (p=0.266).

## DISCUSSION

The prognostic value of CD147 in NSCLC has previously been controversial. In the present meta-analysis, data from 10 studies including 1605 patients were extracted and pooled. The pooled results indicated that CD147 overexpression was associated with shorter OS, the presence of lymph node metastasis and advanced TNM stage. These results suggested that CD147 was a potential marker for poor survival outcome and cancer cell dissemination in NSCLC. CD147 could be a prognostic biomarker that may aid the management of patients with NSCLC. To the best of our knowledge, the current study is the first meta-analysis evaluating the prognostic significance of CD147 in NSCLC.

CD147 is a cell surface glycoprotein that belongs to the immunoglobulin superfamily [27]. CD147 is a multifunctional molecule with various binding partners [28] that is highly expressed on the surface of various cancer cells. CD147 can bind to monocarboxylate transporters (MCTs) and facilitates the proper expression of MCT1 and MCT4 [29]. CD147 is also involved in MCT activation through interaction with CD44 [30]. Furthermore, CD147 facilitates the overexpression of MMPs [31] and vascular endothelial growth factor [32] by tumor cells, which subsequently enhances the migration, proliferation and angiogenesis of cancer cells [28]. Evidence also shows that CD147 expression in cancer cells correlates with chemoresistance and resistance to apoptosis [33–35]. In addition, the overexpression of CD147 is reported to be an unfavorable prognostic marker in various tumors [12, 36–38].

The present meta-analysis pooled inconsistent results for the first time and revealed the prognostic role of CD147 in NSCLC. Interestingly, several other meta-analyses also reported the prognostic value of CD147 in a variety of tumors, including prostate cancer, glioma, and gastrointestinal cancer [8, 39–43]. Huang et al [40] showed that CD147 expression was associated with poor disease-free survival (DFS) and OS in gastrointestinal cancer. Kong et al [41] demonstrated that CD147 was related to higher World Health Organization grading in glioma. In addition, Ye et al [43] also identified a significant association between CD147 and the clinicopathological characteristics of prostate cancer. These findings were in accordance with the results of the present meta-analysis. Therefore, CD147 may serve as a potential prognostic marker across cancer types. Notably, Sienel et al [22] showed that membranous localization of EMMPRIN (CD147) was associated with poor survival in patients with adenocarcinoma in univariate analysis (p=0.03). Moreover, in multivariate analysis, membrane-bound EMMPRIN was the second strongest indicator of poor survival (HR=2.1, 95% CI= 1.0–4.4, p=0.04), compared with the cytoplasmic EMMPRIN staining pattern. This previous study suggested that EMMPRIN staining pattern could be a potential indicator for survival outcomes. A membranous EMMPRIN localization was a potential marker for aggressive cancer phenotype. Further studies are needed to investigate the underlying mechanisms of this phenomenon.

Several limitations of our study should be noted. First, majority of the included studies were from Asian countries. Therefore, the results may be more applicable for Asian patients, and patients with diverse ethnic backgrounds need to be recruited in relevant studies to validate our results. Second, the reported HR and 95% CI instead of individual information from each patient were used to calculate the pooled HR and 95% CI. Third, studies with negative results may be unlikely to be published. Therefore, although Begg's test suggested no significant publication bias, potential selection bias may still exist.

In summary, this meta-analysis demonstrated that the overexpression of CD147 correlated with shorter OS, presence of lymph node metastasis, and advanced TNM stage in NSCLC. CD147 could serve as a potential prognostic marker for NSCLC.

## MATERIALS AND METHODS

### Search strategy

We carried out this meta-analysis following the guidelines of the Preferred Reporting Items for Systematic Reviews and Meta-Analyses Statement [44]. Electronic platforms including PubMed, Web of Science, Embase, and China National Knowledge Infrastructure were systemically searched. The latest search was updated on October 29, 2016. The searched key terms were: (CD147 OR extracellular matrix metalloproteinase inducer OR EMMPRIN OR BASIGIN OR HAb18G) AND (lung cancer or NSCLC or lung carcinoma). Reference lists of relevant studies were also manually checked for additional records.

### Inclusion and exclusion criteria

Eligible literature was required to meet the following inclusion criteria: (1) patients were diagnosed with NSCLC using pathological or histological confirmation; (2) CD147 expression was assessed by immunohistochemical staining (IHC); (3) studies reported the associations between CD147 and OS or clinicopathological factors; (4) studies published in English or Chinese. Studies were excluded based on the following criteria: (1) letters, reviews, meeting abstracts; (2) animal studies; (3) duplicate studies; and (4) studies with insufficient data.

### Data extraction and quality assessment

For each eligible study, the following information was extracted by two independent investigators (XJZ and TT): first author, publication year, study country, number of patients, detection method, mean age, tumor stage, positive rate of CD147, and HR, and 95% CI for OS. If the HR and 95% CI were not directly presented in text, they were calculated according to Tierney's method [45]. Any discrepancies between the two investigators were settled by discussion. The methodological quality of eligible studies was assessed using the Newcastle-Ottawa Scale [46]. The scale evaluated a study based on three aspects: selection, comparability, and outcome, with a score up to 9. Studies with a score of ≥6 were considered to be high quality.

### Statistical analysis

To evaluate the prognostic significance of CD147 expression, pooled HRs, ORs and 95% CIs were calculated. Heterogeneity was assessed using Cochran's Q test [47] and Higgins I-squared statistic [48]. P value of heterogeneity <0.10 or *I*^2^>50% was considered as significant heterogeneity, and the random effect model was adopted; otherwise, the fixed effect model was applied. Sensitivity analysis was conducted to examine the impact of individual studies on the pooled result. Publication bias was tested using Begg's test. All statistical analyses were conducted using Stata version 12.0 (Stata Corp., College Station, TX). P<0.05 was considered statistically significant.
